# Brain-targeting lipid nanoparticles of nicotinamide mononucleotide: preparation, optimization, and characterization

**DOI:** 10.3389/fchem.2026.1759167

**Published:** 2026-06-17

**Authors:** Yuxian Lin, Fengdong Zhao, Jinhui Wang, Songyuge Ping, Kaihan Wu, Yangfang Chen, Xiang Zheng, Hui Xu

**Affiliations:** 1 School of Pharmacy, Key Laboratory of Molecular Pharmacology and Drug Evaluation (Yantai University), Ministry of Education, Collaborative Innovation Center of Advanced Drug Delivery System and Biotech Drugs in Universities of Shandong, Yantai University, Yantai, Shandong, China; 2 The Third Clinical Institute Affiliated to Wenzhou Medical University, Wenzhou People’s Hospital, Wenzhou, Zhejiang, China; 3 Department of Hepatobiliary Surgery, The First Affiliated Hospital of Wenzhou Medical University, Wenzhou, Zhejiang, China

**Keywords:** brain-targeting lipid nanoparticles, drug release, lactoferrin modification, nicotinamide mononucleotide, response surface methodology

## Abstract

Nicotinamide mononucleotide (NMN) has anti-inflammatory, antioxidant, and mitochondrial function-enhancing properties, which demonstrate its significant neuroprotective potential. However, its high water solubility presents challenges with regard to blood-brain barrier (BBB) permeability, prompting extensive research interest. This study used a composite emulsification method combined with response surface methodology to optimize the formulation and preparation process, resulting in the design of lactoferrin (Lf)-modified lipid nanoparticles (LNPs)—NMN-LNPs-Lf. LNPs prepared under optimal conditions exhibited spherical morphology with an average particle size of approximately 147 nm, achieving a drug loading capacity of 5.02%, and a Lf modification rate of 41.8%. Using the mouse brain endothelial cell line 3 (bEnd.3) experimental results demonstrated that NMN-LNPs-Lf significantly enhanced NMN’s BBB permeability without inducing notable cytotoxicity, achieving a BBB permeability rate of 62%. This permeability was markedly higher than that of conventional unmodified NMN-LNPs and NMN active pharmaceutical ingredients (*P* < 0.01). NMN-LNPs-Lf significantly altered the pharmacokinetic and tissue distribution in rats. Under identical dosing regimens, the ${\text{AUC}}_{\left(0-\infty \right)}$ , Vz/F, CLz/F, C_max_, and ${\text{MRT}}_{\left(0-\infty \right)}$ were significantly higher than API (*P* < 0.01), accompanied by selective NMN enrichment in brain tissue. Overall, NMN-LNPs-Lf demonstrated an enhanced capacity for brain-targeting delivery in both *in vitro* cell models and normal rats, suggesting its potential as a therapeutic strategy for neurodegenerative diseases. Future research should incorporate pathological models to conduct in-depth functional and mechanistic evaluations.

## 1 Introduction

β-Nicotinamide mononucleotide (NMN) is a key precursor molecule in mammalian cells. It is catalyzed by specific enzymes to synthesize nicotinamide adenine dinucleotide (NAD^+^), an essential coenzyme for vital biological processes. NAD^+^ is central to numerous redox reactions and is a vital substrate for longevity proteins (SIRT1-7) and poly (ADP-ribose) polymerases (PARPs). It plays a pivotal role in maintaining cellular energy homeostasis, regulating genomic stability, and modulating mitochondrial function and stress responses (Cai et al., 2024). Recent studies have demonstrated that NMN supplementation effectively increases tissue NAD^+^ levels, thereby activating mitochondrial biosynthetic functions and improving pathological phenotypes associated with aging and related diseases (Desai et al., 2025). In models of neurological disorders, NMN exhibits significant neuroprotective properties, alleviating oxidative stress and neuroinflammation by activating the NAD^+^/PARP1/SIRT1 signaling cascade. It also upregulates the expression of the mitochondrial regulator PGC-1α and inhibits neuronal apoptosis (Desai et al., 2025; Lin et al., 2025). *In vitro* studies further confirm NMN’s distinct protective actions on astrocytes, neurons, and myoblasts (Desai et al., 2025; Huang et al., 2023; Lin et al., 2025; Zhu et al., 2023). However, the potential for the clinical application of NMN is limited by its poor pharmaceutical properties: the molecule is highly water-soluble, has a short half-life *in vivo*, is rapidly cleared by metabolism, and has difficulty penetrating the BBB to reach central nervous system (CNS) lesions, resulting in low brain bioavailability (Cai et al., 2024). To overcome these limitations, nanomedicine technologies have been extensively explored in recent years to improve NMN’s fate *in vivo*. Wang et al. developed NMN-loaded liposomes functionalized with angiopep-2 (ANG) and rabies virus glycoprotein peptide. Using an *in vitro* BBB model based on hCMEC/D3 human endothelial cells, they demonstrated that ANG conjugation significantly enhances nanocarrier transport across the endothelium (Wang et al., 2024). Cai et al. developed a lactoferrin-conjugated polydopamine-based nanoplatform (PDA-Lf-NMN) for brain-targeted NMN delivery. Following systemic administration, it significantly increased NAD^+^ levels in the brains of both young and old mice, confirming successful central nervous system (CNS) delivery (Cai et al., 2024). Meanwhile, Zhang et al. synthesized a hydroxyapatite-based NMN nanocomposite (NMN–HAP) via wet chemical precipitation followed by physical adsorption. This nanodelivery system was shown to enhance NMN bioavailability and elevate NAD^+^ levels *in vivo* significantly (Zhang et al., 2023).

Of the various nanocarriers, LNPs are among the most clinically promising delivery systems due to their excellent biocompatibility, biodegradability, high drug loading capacity, sustained-release properties, and low immunogenicity (Desai et al., 2025; Sharma et al., 2025). However, conventional LNPs face two significant challenges: they are rapidly cleared by the reticuloendothelial system under physiological conditions, and only a small proportion of loaded particles can cross the BBB to reach brain lesions effectively (Sharma et al., 2025). To address these issues, researchers have developed strategies to functionalize the surface of nanoparticles, achieving receptor-mediated transcellular transport by conjugating specific targeting ligands (Desai et al., 2025). Lf, an iron-binding glycoprotein within the transferrin family, has received a great deal of attention in recent years. It possesses multiple physiological functions, including immunomodulation, antibacterial activity, and intrinsic neuroprotective effects (Desai et al., 2025). Crucially, Lf binds specifically to the Lf receptor and the transferrin receptor, which are both highly expressed on brain capillary endothelial cells. This enables efficient crossing of the BBB via receptor-mediated endocytosis (Eker et al., 2023). Compared to other targeting ligands (e.g., transferrin, insulin, and apolipoprotein E), Lf offers the advantages of high targeting efficiency, low immunogenicity, abundant sources, and controllable costs (Desai et al., 2025; Sharma et al., 2025). Furthermore, Lf-modified nanocarriers have demonstrated promising potential for delivering drugs to the brain in models of CNS diseases such as Parkinson’s disease and Alzheimer’s disease (Desai et al., 2025; Gomes et al., 2025). Currently, multiple nanoplatforms, including polymeric, inorganic, and lipid nanoparticles, as well as dendrimers, have successfully achieved Lf functionalization, significantly enhancing drug transport efficiency within the brain (Ponduri and Rao, 2026; Guo et al., 2025; De et al., 2025; Moazzam et al., 2026). However, research combining Lf targeting strategies with lipid nanoparticles (LNPs) for NMN-specific brain delivery remains unexplored.

Against this backdrop, this study innovatively constructed NMN-LNPs-Lf. This approach aims to synergistically leverage the sustained-release protective advantages of LNPs with lactoferrin’s active brain-targeting capability by covalently coupling lactoferrin to the surface of DSPE-PEG_2000_-COOH-modified LNPs (Zöller et al., 2024). This overcomes the limitations of NMN’s short half-life and poor BBB permeability, significantly enhancing its bioavailability and neuroprotective efficacy within the CNS. The study systematically optimized the NMN-LNP formulation process and Lf modification conditions using Box–Behnken response surface methodology (RSM). The brain targeting efficiency was evaluated through *in vitro* cellular uptake experiments, *in vivo* pharmacokinetic studies, and tissue distribution analyses. This research provides novel strategies and experimental evidence for the targeted application of NMN in CNS disorders, while also laying a crucial foundation for the wider use of Lf-modified LNPs in neuropharmaceutical delivery.

## 2 Experimental section

### 2.1 Reagents and animals

NMN was supplied by Yuyao Lifespan Health Technology Co., Ltd (China). Soy lecithin and cholesterol were sourced from Shanghai Maclean Biochemical Technology Co., Ltd. DSPE-PEG_2000_ was obtained from Shanghai Bide Pharmaceutical Science and Technology Co., Ltd. DSPE-PEG_2000_-COOH was provided by Shanghai Pengshuo Biotechnology Co., Ltd. Lactoferrin (Lf) was purchased from Shanghai Yuanye Biotechnology Co., Ltd.

The bEnd.3 cells were sourced from Wuhan Punosai Biotechnology Co., Ltd. Healthy male SD rats (300 ± 20 g, SCXK20220006) were supplied by Jinan Pengyue Company. All animal handling procedures complied with the Guide for the Care and Use of Laboratory Animals and were approved by the Animal Experiment Ethics Committee of Yantai University (No. 20241110).

### 2.2 LNPs preparation

LNPs were prepared using the compound emulsion method (Iqbal et al., 2015). Briefly, an appropriate amount of lecithin, cholesterol, and DSPE-PEG_2000_ were dissolved in chloroform to yield a homogeneous solution. Next, an aqueous solution of NMN at a predetermined concentration was added to the lipid solution. The mixture was then subjected to probe sonication using a cell disruptor. An appropriate volume of ultrapure water was then added, and the resulting emulsion was further sonicated to ensure uniform dispersion. Chloroform was subsequently removed by rotary evaporation under reduced pressure. The resultant suspension was filtered through a 0.22-μm polyethersulfone (PES) membrane filter to sterilize and remove aggregates. The final formulation obtained after these steps was designated as NMN-loaded LNPs (NMN-LNPs).

DSPE-PEG_2000_-COOH and DSPE-PEG_2000_ were mixed at a predetermined molar ratio and incorporated into the NMN-LNPs formation. Then, EDC and NHS were added, and the mixture oscillated in a water bath at room temperature for 30 min. An appropriate amount of Lf was then added, followed by further oscillation for 12 h at room temperature. Finally, dialysis for 24 h eliminated unbound Lf and excess EDC and NHS. The final product was designated as Lf-modified NMN-LNPs-Lf after removal of free Lf, EDC, and NHS.

### 2.3 Box–Behnken experimental design

The formulated NMN-LNPs loaded transfersomes were optimized using the Box–Behnken Design (BBD), a tool within RSM. We used a one-way experimental approach to create a three-factor, three-level BBD. The three independent variables were ultrasonic emulsification time (A), oil-to-water ratio (B), and membrane lipid ratio (C), defined as the lecithin-to-cholesterol ratio (Abdallah et al., 2022). Each factor had three levels. The BBD was implemented with Design-Expert software (version 12), and encapsulation efficiency (Y) was the response variable. We derived the optimal formulation conditions from the model fitting results.

### 2.4 Basic characterization

#### 2.4.1 Morphology, particle size, and zeta potential

NMN-LNPs and NMN-LNPs-Lf were prepared repeatedly using the optimal conditions determined by the BBD. The encapsulation efficiencies of these three preparations were then determined. Next, the LNPs samples were diluted 50-fold, and their particle size, polydispersity index (PDI), and zeta potential were measured using a particle analyzer (Delsa Nano C; Beckman Coulter Inc., Brea, CA, United States). To examine the ultrastructure, the samples were further diluted 5-fold with deionized water, and a drop was placed onto a copper grid. The samples were then stained with a 2% sodium phosphotungstate solution for 5 min before being air-dried. The morphology was visualized using a Hitachi H-600 transmission electron microscope (TEM) (Hitachi, Tokyo, Japan).

Residual chloroform in the preparation was detected using an Agilent 6,890 gas chromatograph (GC) coupled with a 5,973 mass selective detector (MSD). For each of the three batches of NMN-LNPs-Lf samples, 1 mL was accurately pipetted and mixed with 1 mL of dichloromethane stock solution for demulsification. After demulsification, the mixture was dissolved in DMSO and then further diluted with purified water. This solution was made up to a final volume of 25 mL in volumetric flasks, ensuring consistent sample preparation for analysis. After sealing and thorough vortexing, the prepared samples were subjected to GC-MSD analysis. Each sample was analyzed in triplicate. Following resealing and thorough mixing, residual organic solvents were quantified based on the peak area ratio of the analyte to the internal standard.

#### 2.4.2 Drug loading and encapsulation efficiency

Drug loading (DL) and encapsulation efficiency (EE) were assessed using High-Performance Liquid Chromatography (HPLC). A 20 µL sample from each NMN-LNPs and NMN-LNPs-Lf solution was diluted 1:10 with methanol, mixed, and vortexed for 1 min to disrupt the emulsion. The mixture was centrifuged at 3,000 rpm for 3 min to separate the phases. The supernatant was collected and analyzed. NMN concentration was quantified with a Waters Alliance E2695 Chromatography System (Waters Corporation, United States) using an Agilent TC-C_18_ column (4.6 × 150 mm, 5 μm) at 30 °C, 1 mL/min flow rate, detection at 265 nm, and a 10 μL injection volume per sample.

$$\text{DL }\left(\%\right)=\frac{W_{\text{NMN}}\;}{\;W_{\text{LNPs}\left(\text{LNPs}-\text{Lf}\right)}}\times 100$$

$$\text{EE }\left(\%\right)=\frac{W_{\text{NMN}}\;}{\;W_{\text{NMN}\;\text{initial}}}\times 100$$

W_NMN_ is the mass of NMN encapsulated in the LNPs or LNPs‐Lf. W_LNPs(LNPs-Lf)_ is the total mass of the freeze-dried LNPs or LNPs‐Lf. W_NMN initial_ is the total mass of NMN initially added during preparation.

#### 2.4.3 Determination of the Lf ligation rate

NMN-Lip-Lf (300 μL) was pipetted into an EP vial and mixed with methanol (400 μL). The resulting mixture was subjected to vortexing for 30 s, after which 200 μL of chloroform was added. Subsequently, the mixture underwent an additional vortexing step for 30 s. Following this, deionized water (100 μL) was added to the solution. The combined mixture was vortexed for another 30 s and then centrifuged at 9,000 rpm for 3 min to remove the upper layer. An additional portion of methanol (400 μL) was then added, followed by a further vortexing period of 30 s and centrifugation at 9,000 rpm for 3 min to obtain a white precipitate.

To investigate Lf connectivity in NMN-LNPs-Lf using the BCA protein assay kit, a standard curve was first established. Bovine serum albumin (BSA) was diluted in PBS (pH 7.4) to a 0.5 mg/mL solution, then further diluted to concentrations of 0, 0.05, 0.1, 0.15, 0.2, 0.3, 0.4, and 0.5 mg/mL. All gradient solutions were incubated at 37 °C for 15 min before measuring absorbance at 562 nm. The NMN-LNPs-Lf precipitate was dissolved in 200 μL PBS, mixed with the BCA working reagent, and incubated for 15 min at 37 °C. Absorbance was measured at 562 nm. Protein content was calculated using the standard curve equation, and conjugation efficiency (CE) was determined by substituting the results into the following equation:

$$\text{CE}\%=\left(1‐\frac{C_{\text{free}}}{C_{\text{total}}}\right)\times 100\%$$

C_free_ is the concentration of free Lf in the solution, C_total_ is the total amount of Lf input.

#### 2.4.4 Stability of lyophilized NMN-LNPs-Lf

The stability of the optimized freeze-dried NMN-LNPs-Lf formulation was evaluated under two storage conditions: 4 °C ± 1 °C and 25 °C ± 2 °C. Tests were conducted at time points of 0, 1, 3, 7, and 14 days in tightly closed containers. Samples were analyzed for particle size, EE, and PDI. The study was conducted in compliance with ICH guidelines.

#### 2.4.5 *In vitro* drug release

The positive dynamic dialysis method was employed for assessing drug release *in vitro*. Three samples each of NMN, NMN-LNPs, and NMN-LNPs-Lf (all 1 mg/mL) were loaded into a dialysis bag (14 kDa) and placed into 15 mL centrifuge tubes with phosphate-buffered saline (PBS, pH 7.2–7.4). The tubes were sealed with clamps and suspended in 200 mL PBS at pH 7.4 in a water bath at 37 °C, rotating at 50 rpm. At specific time points (0.1, 0.3, 0.5, 1, 2, 3, 4, 6, 8, 12, 24 h), 200 μL aliquots were withdrawn and replaced with an equal volume of fresh medium to maintain constant conditions. Drug concentrations and cumulative release rates were measured using a UV spectrophotometer, and dissolution curves for each formulation were plotted accordingly.

### 2.5 Blood-brain barrier permeability measurement

#### 2.5.1 *In vitro* cytotoxicity evaluation

BEnd.3 cells in logarithmic growth were trypsinized and seeded at 1 × 10^4^ cells per well in 96-well plates. After 24 h, culture medium with NMN-LNPs and NMN-LNPs-Lf (at 10, 20, 50, 100, and 200 μg NMN) was added. After a 24 h incubation, cells were rinsed with PBS, and fresh culture medium was added. Then, 10 μL CCK-8 solution was dispensed into each well and incubated for 4 h. The absorbance (A) at 450 nm was measured using a multimode microplate reader (Spark, TECAN, Switzerland). The experiment was repeated three times for reproducibility. Cell viability (CV) was calculated as follows:

$$\text{CV}\left(\%\right)=\frac{A2‐A0}{A1‐A0}\times 100\%$$

A0, A1, and A2 represent the absorbance of the blank, control, and sample, respectively.

#### 2.5.2 BBB model

BEnd.3 cells were inoculated at 1.0 × 10^5^ cells/cm^2^ into the upper chamber of a 12-well Transwell plate. Culture medium (0.5 mL and 1.5 mL) was added to the upper and lower chambers, respectively. The plate was incubated at 37 °C with 5% CO_2_. Resistance differences were monitored using a Cytoresistance Meter (T) between the chamber and the external environment was monitored using a Cytoresistance Meter (T). Using the blank chamber without cell seeding as a reference, the transmembrane electrical resistance (TEER) was calculated as (TR-TO) × A, with TR and TO representing resistance in the cell-seeded and blank chamber, and A as the surface area. A stable TEER value above 200 Ω·cm^2^ indicated successful model construction (Jiang et al., 2019).

The medium solution containing the drug under investigation was introduced into the upper chamber of the Transwell plate. Following a 24 h incubation, HPLC analysis was used to measure drug content in the lower chamber solution. The ratio of the total drug amount detected in the lower chamber to the amount initially added was calculated to assess BBB permeability.

### 2.6 Administration of treatment to rats and collection of biological samples

Sprague Dawley rats were randomly allocated into three groups (n = 6) and administered NMN, NMN-LNPs, or NMN-LNPs-Lf (3 mg/kg NMN equivalent) via tail vein injection. Blood was collected from the orbital venous plexus before administration and at several post-dose intervals (5, 10, 20, 30 min, 1, 2, 4, 8, 12, and 24 h). Plasma was subsequently isolated and cryopreserved at −80 °C for further analysis. Subsequent to a 1-week acclimation period, healthy Sprague Dawley rats were randomly divided into two groups (n = 6) based on body weight. The animals received a single tail-vein injection of either free NMN or NMN-Lip-Lf at a dose of 3 mg/kg (calculated as NMN) and a volume of 10 mL/kg 20 min after the administration of the drug, the rats were euthanized, and tissue samples were collected from the heart, liver, spleen, lungs, kidneys, and brain. Following a thorough cleansing with saline, the samples were meticulously dried using filter paper and weighed. Thereafter, each sample was meticulously collected and stored at −80 °C, where they were reserved for subsequent processing and analysis.

### 2.7 LC-MS/MS for determining NMN concentrations

Tissues were homogenized in four times their volume of saline. For plasma and tissue sample pretreatment, an aliquot of 50 μL of each biosample was placed into a 1.5 mL Eppendorf tube. After this, 100 μL of an internal standard (IS) solution containing ginsenoside Rg1 (200 ng/mL) was added to each sample. Subsequently, 100 μL of a methanol-water mixture (6:4, v/v) was introduced. The mixture was vortexed for 3 min. Plasma samples were centrifuged at 12,000 rpm for 10 min at 4 °C, while tissue homogenates were centrifuged at 12,000 rpm for 15 min at 4 °C. Finally, the supernatant was decanted into a new tube for subsequent analytical processing.

The LC-MS/MS conditions were set as follows. The column used was Accucore™ HILIC (100 mm × 2.1 mm, 2.6 µm). The mobile phase consisted of 0.1% formic acid aqueous solution (phase A) and methanol (phase B). The flow rate was set to 0.3 mL/min with the column temperature maintained at 30 °C. The injection volume was 5 μL. For detection, electrospray ionization (ESI) served as the ion source. The instrument operated with an ion spray voltage of 5500 V in positive ion mode, a capillary temperature of 40 °C, and an air curtain pressure of 25 psi. Data acquisition was performed in Multiple Reaction Monitoring (MRM) mode, monitoring the transitions *m/z* 335.0/123.0 for NMN and *m/z* 801.0/823.5 for the internal standard, ginsenoside Rg1.

### 2.8 Statistics analysis

Pharmacokinetic data were analyzed using DAS 2.0 software to determine pharmacokinetic parameters. Other data were expressed as mean ± standard deviation (Mean ± SD). Statistical comparisons of intergroup data were performed using an independent samples *t*-test in SPSS 23.0 software. A *P*-value <0.05 was defined as statistically significant.

## 3 Results and discussion

NMN has attracted considerable attention as a potential therapeutic agent for neurodegenerative and age-related diseases. To facilitate BBB penetration, this research introduces several methodological and conceptual innovations. In terms of carrier design, we developed a lactoferrin-conjugated NMN nanoparticle using an emulsion-based synthesis approach that was subsequently optimized via Box–Behnken RSM. This approach departs from the conventional use of polydopamine-coated or unmodified liposomal platforms. This hybrid architecture combines the biocompatibility of liposomal carriers with the structural integrity and colloidal stability of solid nanoparticles. Meanwhile, systematic formulation optimization yielded an encapsulation efficiency, representing a significant improvement in drug loading capacity compared with existing NMN delivery systems. Lactoferrin functionalization gave the particles pronounced targeting specificity towards the BBB, as shown by a notable increase in transendothelial transport efficiency in an *in vitro* BBB model compared to the non-targeted control. Comprehensive physicochemical characterization was also performed, including particle size distribution via dynamic light scattering (DLS), zeta potential analysis, *in vitro* release kinetics under physiological conditions, and long-term colloidal stability assessment. *In vivo* biodistribution studies in rodent models corroborated the brain-targeting capability of the engineered nanosystem across multiple experimental dimensions.

### 3.1 LNPs preparation and characterization

#### 3.1.1 Single factor investigation

##### 3.1.1.1 Membrane lipid ratio

**FIGURE 1:**
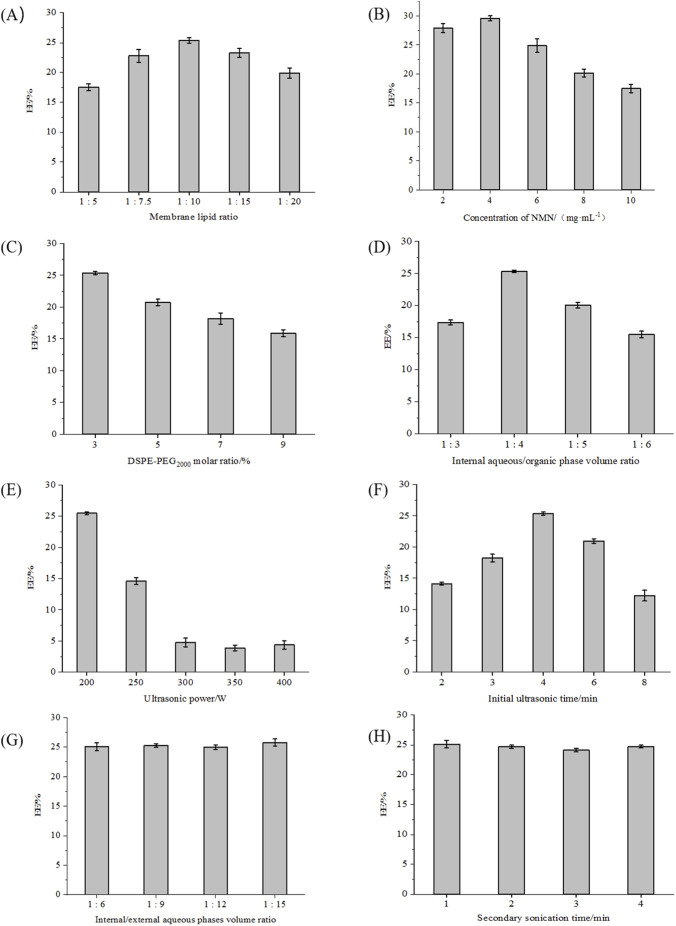
The impact trend of prescription and process factors on the EE of LNPs (mean ± SD, n = 3) **(A)** Membrane lipid ratio; **(B)** Concentration of NMN; **(C)** DSPE-PEG_2000_ molar ratio; **(D)** Internal aqueous/organic phase volume ratio; **(E)** Ultrasonic power; **(F)** Initial ultrasonic time; **(G)** Internal/external aqueous phases volume ratio; **(H)** Secondary sonication time (n = 3).

The effect of membrane lipid ratios (1:5, 1:7.5, 1:10, 1:15, 1:20) on the EE of NMN-LNPs was studied. EE increased as the membrane lipid ratio decreased and cholesterol content increased, peaking at a ratio of 1:10 (Figure 1A). NMN-LNPs consisted of a phospholipid bilayer, and adding cholesterol stabilizes the LNPs. Excessive cholesterol made the LNPs' membrane more rigid, leading leakage of the inner aqueous phase and reducing EE. Thus, a membrane-to-lipid ratio of 1:10 was optimal for this experiment.

##### 3.1.1.2 Concentration of drug solution

Drug solution concentrations were set at 2, 4, 6, 8, and 10 mg/mL to observe their effect on NMN-LNPs EE. EE peaked at 4 mg/mL (Figure 1B). The EE of LNPs increased with higher drug concentrations until saturation; beyond this point, EE declined as concentration rose. Consequently, 4 mg/mL was identified as optimal for this experiment.

##### 3.1.1.3 DSPE-PEG_2000_ molar ratio

A one-step method of LNPs PEGylation was employed, utilizing DSPE-PEG_2000_ as the long-circulating agent. Briefly, the long-circulating NMN-LNPs were generated through membrane formation using lecithin and cholesterol. DSPE-PEG_2000_ was then incorporated to inhibit the phagocytosis of LNPs by macrophages in the bloodstream after intravenous administration. As a result, this enhanced the stability of the particles. However, one challenge was that excessive DSPE-PEG_2000_ might cause drug leakage from NMN-LNPs (Ren et al., 2018). To address this, experiments were conducted with molar ratios of 3%, 5%, 7%, and 9% (Figure 1C). Results showed that the EE of LNPs diminished as the proportion of DSPE-PEG_2000_ increased. This decline was attributed to a reduction in the LNPs particle size and a weakening of the lamellar structure at higher DSPE-PEG_2000_ ratios. Specifically, a smaller particle size restricted drug encapsulation capacity, while weakened lamellar integrity contributed to drug leakage. Together, these factors led to a reduced EE (Sriwongsitanont and Ueno, 2004). Ultimately, a DSPE-PEG_2000_ molar ratio was selected that achieved an EE of 3%. This molar ratio has been confirmed by another study as optimal for DSPE-PEG_2000_ (Haghiralsadat et al., 2018).

##### 3.1.1.4 Dosage of DSPE-PEG_2000_-COOH

This experiment examined the effect of varying molar ratios between DSPE-PEG_2000_-COOH and DSPE-PEG_2000_ (5%, 10%, 15%, 20%, and 30%) on Lf grafting levels and consequent impact on targeting efficiency. Selection of the 10% molar ratio for DSPE-PEG_2000_-COOH was based on achieving an approximately 40% linkage rate for Lf, while maintaining consistent targeting efficacy across reactive LNPs. This ratio offered a balance between Lf conjugation and formulation stability. The final formulation parameters for NMN-LNPs were set as follows: a membrane-lipid ratio of 1:10; drug concentration of 4 mg/mL; DSPE-PEG_2000_ molar ratio at 3%; and DSPE-PEG_2000_-COOH prescribed at 10%.

##### 3.1.1.5 Organic phase to internal aqueous phase volume ratio

We maintained the volume of the internal aqueous phase constant and observed the effects of various volume ratios of the internal aqueous phase to the organic phase (1:2, 1:3, 1:4, 1:5, and 1:6) on EEs. It was observed that the EE reached its maximum at a volume ratio of 1:4 (Figure 1D).

##### 3.1.1.6 Ultrasonic power and the initial ultrasonic emulsification time

The ultrasonic power settings used in this experiment were 200, 250, 300, 350, and 400 W. Observations of the drug EE indicated a gradual decrease in its concentration as the ultrasonic power increased (Figure 1E). At ultrasonic power levels below 200 W, the emulsification effect was suboptimal. Conversely, excessively high ultrasonic power resulted in leakage of the aqueous phase from the LNPs, thereby diminishing the EE. For this reason, an ultrasonic power setting of 200 W was determined to be optimal for the LNPs study.

The EE of the LNPs was evaluated at various ultrasonic emulsification times (2, 3, 4, 6, and 8 min). It was observed that the EE initially increased before subsequently decreasing. The optimal duration for the first ultrasonic emulsification was determined to be 4 min. This phenomenon was reflected in the heat generated during ultrasonication. As the emulsification time prolonged, the system temperature also rose, potentially leading to a phase transition in the LNPs membrane material and accelerating oxidation processes. These factors ultimately contributed to a reduction in drug EE (Figure 1F).

##### 3.1.1.7 Other factors

The EE of LNPs was evaluated by volume ratios towards internal aqueous phase to external aqueous phase (1:6, 1:9, 1:12, and 1:15). It was found that the EE changed slightly in the different levels of volume ratios. To prevent the emulsion from being dried due to insufficient volume during the subsequent vacuum evaporation process, the volume ratio of the inner aqueous phase to the outer aqueous phase was finally determined to be 1:9 (Figure 1G).

The research team demonstrated the experimental findings concerning the duration of secondary sonication emulsification (Figure 1H). Variations in the duration of secondary sonication emulsification did not significantly affect the EE of LNPs. Considering the actual experimental conditions and overall efficiency, a 1-min duration was ultimately determined as optimal for secondary sonication emulsification.

Meanwhile, LNPs underwent decompression evaporation at 32 °C for 10 min, achieving the highest EE. When the decompression evaporation temperature was too low or too high, or the time was either too short or too long, a reduction in drug EE was observed.

#### 3.1.2 Box-Behnken design

A Box-Behnken experimental design, adhering to the principles of RSM, was utilized to generate a total of 17 test points. The associated response variables are tabulated in Table 1. Regression analysis was carried out using Design-Expert 13. The regression equation between the independent and dependent variables was derived from the fitting process:

$$Y=29.52+0.9A+0.6375B‐1.24C+0.675\text{AB}‐0.375\text{AC}‐0.3\text{BC}‐1.76A^{2}‐1.54B^{2}‐2.33C^{2}\left(R^{2}=0.994\right)$$

**TABLE 1:** Box-Behnken design and results of formulation and process optimization.

No.	Initial sonication time, min (A)	Internal organic/aqueous phase ratio (B)	Membrane-to-lipid ratio (C)	Entrapment efficiency, % (Y)
1	4	5	8	27.8
2	4	4	10	29.1
3	4	4	10	29.4
4	4	4	10	29.7
5	6	3	10	25.7
6	2	3	10	25.5
7	4	3	8	25.9
8	6	4	8	28.1
9	6	5	10	28.3
10	2	4	8	25.3
11	2	4	12	23.5
12	6	4	12	24.8
13	4	4	10	29.6
14	2	5	10	25.4
15	4	5	12	24.8
16	4	3	12	24.1
17	4	4	10	29.8

The regression model for EE was highly significant (*P* < 0.0001), while the lack-of-fit term was non-significant (*P* = 0.6507), indicating good model adequacy. Furthermore, the following factors exhibited a statistically significant impact on EE (*P* < 0.05): A, B, C, AB, AC, BC, A^2^, B^2^, and C^2^. As shown in Table 1, both the ultrasonic emulsification time and the membrane-lipid ratio significantly affected the EE of NMN LNPs. In alignment with actual clinical practice and experimental requirements, the aim was to minimize LNPs particle size and maximize EE. Subsequently, the regression model was further analyzed to quantify the degree of interaction among the three factors and to visualize how this interaction influences the desirability of EE.

**FIGURE 2:**
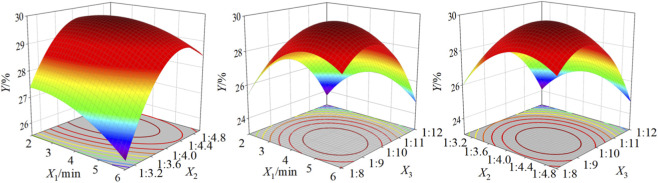
Response surface plots of DOE showing the effects of formulation on entrapment efficiency (Y) of NMN-LNPs. X_1_, X_2_, and X_3_ represented the factors of initial sonication time, internal organic/aqueous phase ratio, and phospholipid/cholesterol ratio, respectively.

The elliptical shape and steep slope of the response surface suggested that there were significant interactions between the two factors. Specifically, the highest response values were located within the selected range for each factor, as indicated by the highest points marked on the response surfaces. The 3D plots of the overall response surfaces were all bell-shaped with a relatively gentle slope and a high point. In addition, the contour lines of interaction were sparse and nearly circular (Figure 2). This finding suggested a statistically significant interaction among ultrasonic emulsification time, oil-to-water ratio, and membrane-to-fat ratio (*P* < 0.05), which was consistent with the results of the analysis of variance (ANOVA). Overall, the oil-water and membrane-fat ratios had a greater effect on the EE, while ultrasonic emulsification time had a lesser effect.

Based on the expected constraints on the range of independent variables, and the goals to minimize and maximize the EE, the three best factors for NMN-LNPs were: X_1_ = 4.5 min, X_2_ = 4.3, and X_3_ = 9.5. The EE (Y) was 29.3%. The difference between this value and the one predicted by RSM was just 0.6%, showing that the RSM model was reliable.

### 3.2 Validation of the optimal process

**FIGURE 3:**
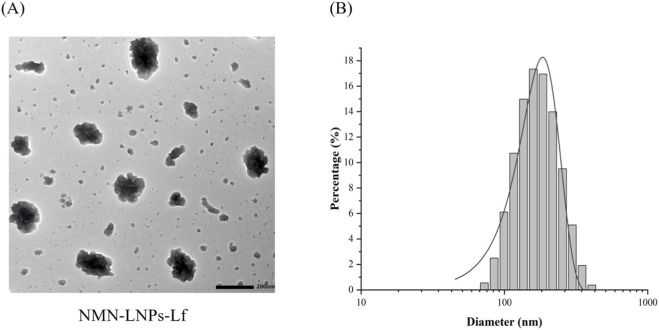
Transmission electron microscopy image **(A)** and DLS size distribution curve **(B)** of NMN-LNPs-Lf.

To further validate the optimal prescription, NMN-LNPs and NMN-LNPs-Lf were prepared. Specifically, the DL and EE of NMN-LNPs were 5.92% ± 0.06% and 29.26% ± 0.55%, respectively, while those of NMN-LNPs-Lf were 5.02% ± 0.04% and 25.03% ± 0.31%. Notably, both LNPs exhibited a uniform particle size distribution without aggregation. Furthermore, transmission electron microscopy (TEM) analysis revealed that NMN-LNPs had a smooth spherical surface. In contrast, NMN-LNPs-Lf presented a spherical shape with a concave and convex surface following Lf modification (Figure 3). The values of LNPs were obtained via DLS as follows (Figure 3): (1) NMN-LNPs: Particle size: 159.85 ± 0.78 nm; PDI: 0.25 ± 0.04; Zeta potential: (10.40 ± 0.88) mV; (2) NMN-LNPs-Lf: Particle size: 147.4 ± 5.49 nm; PDI: 0.26 ± 0.05; Zeta potential: (8.87 ± 0.41) mV. Additionally, the lactoferrin modification rate of NMN-LNPs-Lf was 41.8% ± 0.24%. Since the PDI of the optimized formulation was less than 0.3, these results confirm that NMN-LNPs and NMN-LNPs-Lf exhibited a uniform particle size distribution and good homogeneity (Jacob et al., 2020). Moreover, the physical stability of these two LNPs was evaluated by measuring the zeta potential. The negative charge on the surface of the LNPs might originate from the particle’s own surface or the surfactants adsorbed on the surface, and such surface charge was beneficial to maintaining the stability of the LNPs (Kesisoglou et al., 2007).

The physical property data for LNPs are summarized in Table 2. As shown by DLS measurements, NMN-LNPs had an average diameter below 160 nm, which decreased by approximately 10 nm following Lf modification. This observed reduction correlated with the increased surface complexity of Lf-modified LNPs, suggesting that Lf modification effectively alters nanoparticle surface architecture. The brain delivery LNPs were engineered to be less than 200 nm in size to promote endocytosis by brain capillary cells, supporting their suitability for brain-targeted delivery (Olivier et al., 2002). Furthermore, NMN-LNPs and NMN-LNPs-Lf particles both exhibited relatively negative zeta potential values, which contributed to maintaining the stability of lipid nanoparticles, especially in biological settings (Leyva-Gómez et al., 2015).

**TABLE 2:** Main characterization of NMN LNPs and LNPs-Lf (n = 3).

Investigated Items	NMN-LNPs	NMN-LNPs-Lf
Particle size (nm)	159.85 ± 0.78	147.40 ± 5.49
PDI	0.25 ± 0.04	0.26 ± 0.05
Zeta potential (mV)	-(10.40 ± 0.88)	-(8.87 ± 0.41)
EE (%)	29.26 ± 0.55	25.03 ± 0.31
DL (%)	5.92 ± 0.06	5.2 ± 0.04

Residual chloroform was determined as 21.0 μg/g, 23.0 μg/g, and 19.6 μg/g for the three batches of NMN-LNPs-Lf, respectively. The relative standard deviation (RSD) was less than 7%. All levels were below the threshold limit of 0.006% in Table 3. The results conformed to the Chinese Pharmacopeia specifications for chloroform residues.

**TABLE 3:** Quantification of residual organic solvents of NMN-LNPs-Lf.

Batch No.	Chloroform/%	RSD/%
20250801	0.002101 ± 0.000120	5.57
20250802	0.002300 ± 0.000089	3.89
20250803	0.001963 ± 0.000132	6.74

### 3.3 Stability studies

Stability experiments of NMN-LNPs-Lf were performed over a total of 14 days, with measurements taken at 0, 1, 3, 7, and 14 days under two different conditions. Particle size, DL, and PDI were evaluated in relation to the influence of stability conditions, and the data were presented in Table 3. When stored under refrigerated conditions (4 °C ± 1 °C), the particle size increased slightly from the initial 147.42 ± 5.31 nm to 151.20 ± 4.35 nm on day 17. At room temperature (25 °C ± 2 °C), a similar increasing trend was observed over the 14-day period, with particle size rising from 148.27 ± 3.07 nm to 155.04 ± 5.28 nm. Over 14 days, average particle sizes increased by about 3.78% and 6.77% without precipitation, and DL declined by 0.16% and 0.15%, indicating acceptable stability at 4 °C and room temperature, respectively (Table 4). All NMN-LNPs-Lf were without precipitation.

**TABLE 4:** The stability study of NMN-LNPs-Lf formulation under different conditions (n = 3).

Temperature	Days	Particle size (nm)	DL%	PDI
4 °C ± 1 °C	0	147.42 ± 5.31	5.01 ± 0.13	0.26 ± 0.02
	1	145.00 ± 5.16	4.98 ± 0.15	0.27 ± 0.02
	3	149.19 ± 7.42	5.03 ± 0.21	0.25 ± 0.02
	7	148.69 ± 6.06	4.89 ± 0.20	0.28 ± 0.03
	14	151.20 ± 4.35	4.85 ± 0.13	0.28 ± 0.02
25 °C ± 2 °C	0	148.27 ± 3.07	5.03 ± 0.25	0.27 ± 0.02
	1	149.11 ± 2.89	4.97 ± 0.23	0.25 ± 0.01
	3	152.43 ± 2.44	4.94 ± 0.17	0.27 ± 0.02
	7	153.04 ± 2.35	4.99 ± 0.13	0.28 ± 0.02
	14	155.04 ± 5.28	4.88 ± 0.18	0.29 ± 0.02

The minor increase in particle size at higher temperatures might be attributed to gradual instability over time. Specifically, lower surface coverage led to size enlargement due to Ostwald ripening (Guo et al., 2024). Meanwhile, both DL and PDI underwent only slight changes with insignificant amplitudes. These results indicated that the NMN-LNPs-Lf formulation remained relatively stable at 4 °C and room temperature.

### 3.4 *In vitro* drug release and its mechanism

**FIGURE 4:**
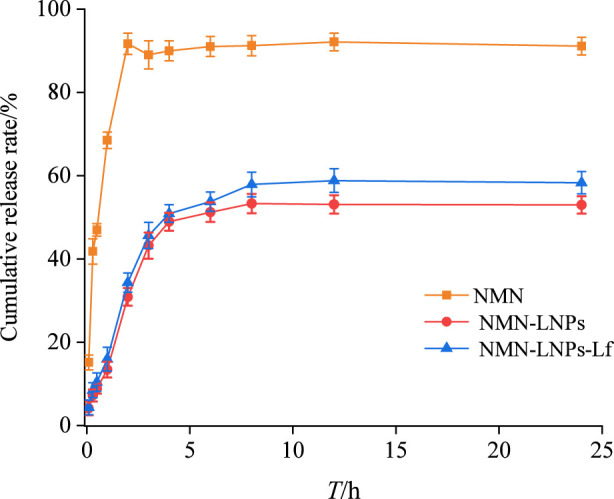
*In vitro* release studies of NMN from free NMN, NMN-LNPs, and NMN-LNPs-Lf (n = 6).

The *in vitro* release profiles of NMN from different formulations in PBS (pH 7.4) at 37 °C are presented in Figure 4. Free NMN exhibited rapid release, with a cumulative release rate reaching 91.67% within 2 h. In contrast, both NMN-LNPs and NMN-LNPs-Lf at 2 h showed a sustained release pattern under the same conditions. At the 2 h time point, the cumulative release rates were 34.34% ± 2.34% and 30.91% ± 2.14%, respectively, gradually rising to 57.89% and 53.31% by 8 h. No significant initial burst release was observed, suggesting good formulation stability during the preparation process.

To investigate the release mechanism of the NMN-LNPs-Lf formulation in this simplified medium, the release data were fitted to four common kinetic models using Origin 2024 software.

- Zero-order Model: Mt = 0.67t + 21.03 (R^2^ = 0.3607);
- First-order Model: Mt = 54.05 (1-e^−0.43t^) (R^2^ = 0.9976);
- Higuchi Model: Mt = 1.15t^1/2^ + 12.22 (R^2^ = 0.6098);
- Ritger-Peppas Model: Mt = 21.25t^0.27^ (R^2^ = 0.7458).

The first-order model showed the highest correlation coefficient (R^2^ = 0.9976), suggesting that the release of NMN from the NMN-LNPs-Lf in PBS follows first-order kinetics, where the release rate was proportional to the remaining drug concentration. This kinetic behavior is characteristic of a concentration-dependent diffusion-controlled process. The observed sustained release can be further attributed to the hydrated barrier formed by DSPE-PEG_2000_ at the LNPs’ surface, which effectively slows the diffusion of NMN from the lipid bilayer into the surrounding medium.

From a formulation design perspective, NMN-LNPs-Lf is engineered to exhibit pH-responsive release behavior via the acid-labile hydrazone bond linking DSPE and PEG. Under physiological conditions (pH 7.4), this bond remains stable, ensuring the integrity of the liposomal structure and protecting the encapsulated NMN. Upon receptor-mediated endocytosis into brain capillary endothelial cells, the acidic microenvironment of endosomes/lysosomes (pH 4.5–5.5) rapidly hydrolyzes the hydrazone bond (Kanamala et al., 2018). This leads to the detachment of the PEG shielding layer and the destabilization of the lipid bilayer. This structural perturbation facilitates the rapid release of NMN from the nanocarriers, enabling its therapeutic action. However, it should be noted that the *in vitro* release study was conducted only in PBS at pH 7.4, which does not trigger this pH-responsive mechanism. Therefore, the observed sustained release under neutral conditions primarily reflects the formulation’s passive, diffusion-controlled behavior rather than its active, pH-triggered release capacity.

Nevertheless, the study initially characterized the release profile in PBS (pH 7.4) only at 37 °C. This simple buffer system does not fully replicate the complex physiological environment encountered *in vivo*. Factors such as serum proteins, metabolic enzymes, and pH fluctuations could significantly impact nanoparticle stability and release kinetics in physiological environments, but were not addressed in this study. Therefore, these *in vitro* findings should be interpreted as preliminary evidence that the formulation possesses sustained-release capabilities. Future studies should include release experiments under acidic conditions and in the presence of serum proteins to validate the pH-responsive release mechanism and better mimic the *in vivo* environment, respectively.

### 3.5 Blood-brain barrier permeability

**FIGURE 5:**
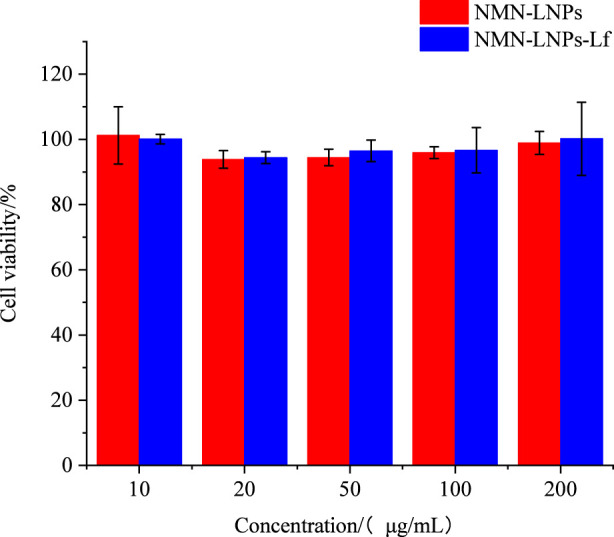
*In vitro* cytotoxicity of NMN-LNPs and NMN-LNPs-Lf (n = 3).

The results of the cytotoxicity experiments are displayed in Figure 5. NMN, at concentrations ranging from 10 to 200 μg/mL, along with NMN-LNPs and NMN-LNPs-Lf, did not exhibit any significant inhibitory or destructive effects on the fundamental physiological functions of bEnd.3 cells, including growth and metabolism. The cytocompatibility was favorable, with no obvious toxicity observed, as NMN exerted minimal interference with cellular physiological activities at specific concentrations. The encapsulation of NMN by LNPs and Lf further enhanced its safety profile. Therefore, these findings ensured that subsequent experiments were conducted under relatively safe conditions, allowing for a more accurate evaluation of the biological effects of NMN-LNPs and NMN-LNPs-Lf. Based on these experimental findings, a dose concentration of 150 μg/mL was selected for subsequent studies, as it supported normal cell growth and metabolism while meeting the drug concentration requirement for further experiments.

**FIGURE 6:**
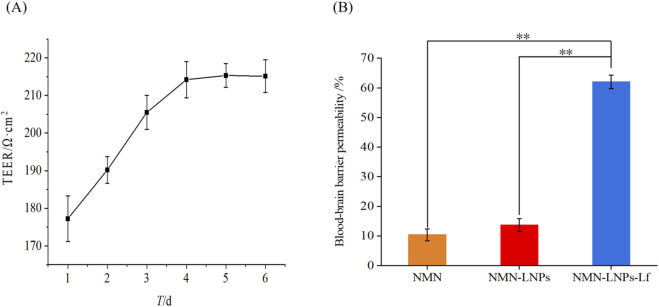
Brain targeting evaluation of NMN formulations in rats: **(A)** Time curve of TEER values; **(B)** BBB permeability of free NMN, NMN-LNPs, and NMN-LNPs-Lf (n = 3, **P < 0.01 vs. free NMN and NMN-LNPs groups).

An *in vitro* BBB model was constructed using bEnd.3 cells to evaluate the comparative permeability of various formulations. As shown in Figure 6A, the model achieved a TEER of 214.20–215.32 Ω·cm^2^ by day four to six post-seeding and maintained stable TEER values thereafter-indicating formation of a functionally intact endothelial monolayer suitable for quantitative BBB permeability assessment. Following validation, NMN (150 μg/mL) was applied to the apical compartment of the Transwell® system. After 24 h of incubation under physiological conditions (37 °C, 5% CO_2_), NMN concentrations were quantified via HPLC to calculate the apparent permeability coefficient. All experiments were performed in triplicate, and TEER measurements were recorded before and after each experiment to confirm monolayer integrity. As summarized in Figure 6B, the transport rates for free NMN, NMN-LNPs, and NMN-LNPs-Lf were 10.37% ± 1.97%, 13.71% ± 2.17%, and 62.03% ± 2.30%, respectively. The NMN-LNPs-Lf formulation exhibited a 3.52-fold increase in BBB permeability compared to free NMN and a 4.98-fold increase relative to NMN-LNPs, with both differences being statistically significant (*P* < 0.01).

Upon Lf conjugation, LNPs permeation across the BBB was markedly improved. The NMN-LNPs-Lf system showed a near 5-fold increase in permeation rate compared to free NMN, demonstrating robust transmembrane capacity. The BBB surface abundantly expresses Lf receptors, which facilitate drug entry into brain tissue via a receptor-mediated, unidirectional transport process involving endocytosis (Mazibuko et al., 2015). This transport modality is advantageous for engineered carriers designed to cross the BBB and penetrate brain tissue.

Lf modification enhanced the uptake rate of drugs by cerebral tissues. Furthermore, Lf exhibited inherent biocompatibility and anti-inflammatory properties (Cai et al., 2024). These characteristics mitigate pro-inflammatory cytokine secretion from glial cells and exert beneficial effects in inflammatory environments associated with neurological disorders such as Parkinson’s disease (Cai et al., 2024; Ryskalin et al., 2021). Thus, Lf modification substantially improved the ability of the encapsulated, water-soluble drug NMN to reach its targeted delivery within brain tissues, underscoring its promising potential for targeted CNS delivery.

Previous research has demonstrated that receptor-mediated transcytosis (RMT) of Lf across the blood-brain barrier (BBB) is a prerequisite for its key effects (Sharma et al., 2025). Lipoprotein receptor-related protein 1 (LRP1) is a receptor that facilitates both transcytosis and endocytosis of Lf at the BBB. It is highly expressed in brain microvascular endothelial cells, neurons, glial cells, and various tumor cells (Mancinelli et al., 2018; Sirvi et al., 2025). Furthermore, emerging evidence indicates that LRP1 also mediates the regulatory roles of Lf in neural iron homeostasis and antioxidant defense (Rascón-Cruz et al., 2024). Consequently, the NMN-LNPs-Lf nanocarrier system developed in this study has significant potential for translational research, particularly with regard to neurodegenerative disorders such as Parkinson’s and Alzheimer’s diseases, as well as tumor-targeted therapy within the CNS.

### 3.6 *In vivo* drug release and tissue distribution

#### 3.6.1 Plasma pharmacokinetic properties

**FIGURE 7:**
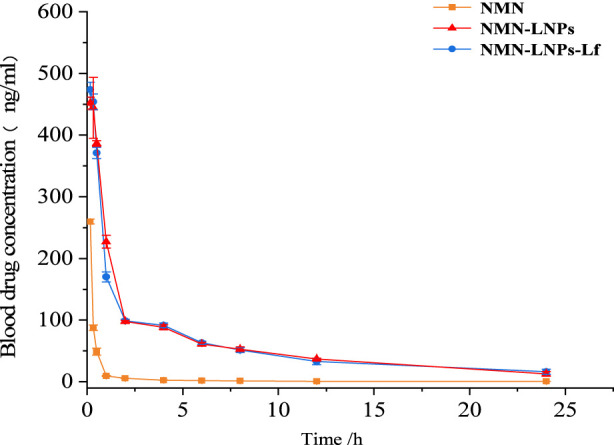
Concentration–time profiles in blood following intravenous administration of NMN, NMN-LNPs, and NMN-LNPs-Lf (n = 6).

The PK results of this study indicate that no significant absorption phase was observed in the NMN, NMN-LNPs, or NMN-LNPs-Lf groups following tail vein injection, which is consistent with the direct entry into the bloodstream characteristic of intravenous administration. As shown in Figure 7, compared to the NMN group, the plasma concentration-time curves for the NMN-LNPs and NMN-LNPs-Lf were nearly identical and remained significantly higher throughout the entire detection period. This phenomenon can be explained by the PK advantages of the nanoparticle formulations: First, upon entering the bloodstream, free NMN readily binds to plasma proteins or is rapidly recognized and cleared by the mononuclear phagocyte system, leading to a rapid decline in blood concentration. In contrast, encapsulation within LNPs provides NMN with a physical barrier, effectively preventing non-specific binding to plasma proteins and rapid recognition by the immune system, thereby prolonging the drug’s residence time in the bloodstream. Second, the nanoscale particle size of LNPs further reduces the likelihood of capillary retention by blood vessel walls and adsorption by plasma proteins (Sakai-Kato et al., 2019), ensuring colloidal stability in plasma and thus maintaining higher blood concentrations.

Compared to the NMN group, the NMN-LNPs and NMN-LNPs-Lf groups demonstrated significant increases in ${\text{AUC}}_{\left(0-\infty \right)}$ , with values 10.23-fold and 10.04-fold higher, respectively. This highlighted the critical role of the LNP formulation in enhancing NMN exposure. Simultaneously, the C_max_ values in both nanoparticle groups were significantly increased by 66.46% and 65.04%, respectively. This further confirmed that LNPs effectively shield NMN from rapid metabolism, thereby sustaining higher plasma concentrations in the circulatory system, a direct reflection of their sustained-releasing and prolonging circulation properties. Moreover, the ${\text{MRT}}_{\left(0-\infty \right)}$ in the NMN-LNPs-Lf group was approximately 5.02-fold and 5.29-fold longer than that in the NMN and NMN-LNPs groups, respectively (*P* < 0.01) (Table 5). This suggested that surface modification with Lf may further delay its *in vivo* elimination, potentially through specific receptor-mediated interactions or alterations in the nanoparticle surface properties.

**TABLE 5:** Pharmacokinetic parameters of NMN in whole blood following tail vein injection of NMN, NMN-LNPs, and NMN-LNPs-Lf.

Parameter	NMN	NMN-LNPs	NMN-LNPs-Lf
${\text{AUC}}_{\left(0-\infty \right)}$ (μg/L*h)	138.94 ± 4.33	1560.82 ± 4.42**	1533.47 ± 134.53**
t1/2z (h)	3.03 ± 0.13	6.42 ± 0.24*	6.88 ± 2.66*
Vz/F (L/kg)	0.09 ± 0.00	17.81 ± 0.63**	19.12 ± 5.66**
CLz/F (h/kg)	0.02 ± 0.00	1.92 ± 0.01**	1.97 ± 0.17**
C_max_ (μg/L)	294.99 ± 17.64	491.05 ± 29.64**	486.85 ± 23.86**
${\text{MRT}}_{\left(0-\infty \right)}$ (h)	1.24 ± 0.03	8.04 ± 0.97	9.32 ± 1.72**##

* *P <* 0.05, **P < 0.01 vs. NMN, group; #*P <* 0.05; ##*P <* 0.01 vs. NMN-LNPs, group.

Notably, this study observed substantial increases in Vz/F and CLz/F for the NMN-LNPs and NMN-LNPs-Lf groups compared to the free drug group. Under conventional PK principles, elevated clearance (CL) typically indicates faster drug elimination from plasma, often associated with reduced systemic exposure. However, in nanomedicine research, alterations in these parameters require careful interpretation in the context of formulation characteristics. The marked elevation in CLz/F does not represent accelerated clearance of the active pharmaceutical ingredient itself but is intrinsically linked to the substantial increase in Vz/F. The dramatic expansion of Vz/F, approximately 200-fold, reflected the profound alteration in NMN biodistribution conferred by LNP encapsulation, facilitating its distribution from the central plasma compartment into peripheral tissues rather than confinement to the bloodstream. According to the fundamental PK equation (CL = k × V), if the elimination rate constant (k) remains unchanged, an expansion of the volume of distribution (V) will mathematically result in a corresponding increase in CL. This phenomenon has been well documented in nanoparticle formulation studies. Multiple studies indicate that factors such as nanoparticle dosage, particle size, and surface modifications can alter the volume of distribution, thereby influencing key parameters such as CL and MRT (Javidi et al., 2019; Sun et al., 2020; Zabala-Gutierrez et al., 2025). Therefore, a comprehensive evaluation of these interrelated metrics is essential in PK studies.

Although substantial increases in CLz/F and Vz/F were observed, this primarily reflects the successful alteration of NMN’s *in vivo* disposition by LNPs—shifting from rapid clearance of the free form to extensive distribution and slow release of the encapsulated form. Tang et al. emphasized that true therapeutic persistence depends not only on carrier longevity in circulation but also on the spatiotemporal distribution of the active drug at the target site. They advocated shifting from a narrow focus on prolonging circulation time toward a comprehensive analytical framework for regulating the entire delivery process (Tang et al., 2024). While traditional PK parameters such as half-life and AUC remain widely adopted as primary indicators of nanomedicine performance, the PK findings from this study align with this evolving concept and provide a robust foundation for subsequent in-depth investigations into tissue targeting and pharmacodynamics.

In summary, encapsulation within LNPs and surface modification with lactoferrin successfully generated an NMN delivery system with sustained-release properties. Significant improvements in key PKs (AUC, Cmax, and MRT) confirm that this nanoformulation effectively protects NMN from rapid metabolism, enabling prolonged circulation and sustained release in plasma. The phospholipid bilayer of LNPs serves as a critical barrier, slowing drug leakage and release rate to extend therapeutic duration. Notably, the plasma half-life of NMN-LNPs-Lf was extended to approximately 7 h, indicating stable and sustained drug delivery. This highlights its potential utility in therapeutic areas requiring long-term maintenance dosing, such as neurodegenerative diseases.

#### 3.6.2 Tissue distribution

**FIGURE 8:**
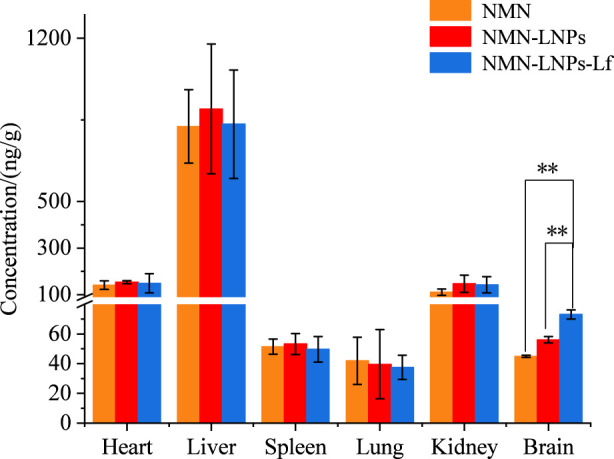
Tissue distribution of NMN in rats after administration of free NMN, NMN-LNPs, and NMN-LNPs-Lf in rats (n = 6, **P < 0.01 vs. both free NMN and NMN-LNPs groups).

As presented in Figure 8, 20 min post tail vein injection, the three NMN dosage forms (NMN, NMN-LNPs, and NMN-LNPs-Lf) were primarily distributed in the liver, kidneys, and heart. The concentrations of NMN in these tissues were comparable across all groups, with no significant differences observed. However, a gradual increase in drug concentration was noted in brain tissue for all three formulations. Notably, the NMN-LNPs-Lf formulation significantly enhanced drug absorption in brain tissue compared to the other two groups (*P < 0.01*), while slightly diminishing NMN absorption in the heart, liver, spleen, lungs, and kidneys. These findings confirmed that NMN encapsulated in LNPs, followed by Lf modification, substantially improves its intracerebral targeting. Lf modification facilitates transport of NMN-LNPs-Lf across the BBB via transcytosis through brain endothelial cells into the brain parenchyma (Qiao et al., 2012; Shan, 2012). This mechanism prevented the NMN degradation in plasma and bypassed efflux transporter activity, prolonging clearance in brain tissue. Consistent with our cellular uptake findings, this enhanced ability to target the brain is likely due to an additional protein-associated transport mechanism (Song et al., 2017). Notably, Lf receptors were overexpressed on BBB endothelial cells, allowing Lf to undergo receptor-mediated transcytosis without intraendothelial degradation (Desai et al., 2025). Therefore, Lf modification improves targeting efficiency and increases the availability of the drug for neural tissue uptake. The enhanced delivery efficiency of the NMN-LNPs-Lf complex, which led to higher drug concentrations, was due to the binding of Lf to its receptor on neuronal cells. It has been suggested that intravenous administration facilitates the more efficient delivery of therapeutic agents to cerebral regions.

The study demonstrates that Lf-modified LNPs apparently enhance the brain accumulation of NMN and exhibit strong BBB penetration capability. These findings align with our initial design hypothesis: Lf modification enables specific binding to the overexpressed lactoferrin receptor (LfR) on brain capillary endothelial cells, thereby facilitating RMT and achieving targeted brain delivery (Desai et al., 2025). However, while the current results are highly consistent with an LfR-mediated uptake mechanism, they provide only indirect evidence and have not yet definitively established the specificity of this process. Although brain uptake in the NMN-LNPs-Lf group was significantly higher than that in both the free NMN and unmodified NMN-LNPs groups, we cannot exclude potential nonspecific contributions to BBB penetration arising from Lf-induced changes in nanoparticle physicochemical properties, such as surface charge, hydrophobicity, or the formation of a protein corona (Cong et al., 2025). Specifically, Lf surface modification may promote nonspecific adsorption of LNPs onto endothelial cells, thereby enabling brain entry via adsorption-mediated endocytosis.

### 3.7 Limitations and future directions

The limitations of the study must be acknowledged, as they will inform future research directions. (1) The integrity of the BBB model had not been experimentally validated; future studies will use immunofluorescence staining for tight junction proteins (ZO-1 and occludin) to address this. (2) Comparative release experiments under acidic conditions (e.g., pH 5.5, simulating the lysosomal environment) were not performed, leaving the validation of the pH-responsive release behavior and mechanism to be conducted in future release studies across different pH conditions. (3) Further experimentation is required to differentiate between specific Lf binding and non-specific adsorption. (4) There is an absence of direct pharmacological or molecular evidence confirming LfR involvement. It is therefore recommended that future investigations incorporate competitive inhibition strategies in an *in vitro* BBB model, including free ligand competition, anti-LfR antibody blockade, and LfR gene silencing/knockout. Notwithstanding these mechanistic limitations, the Lf-modified LNPs developed herein unambiguously enhance the brain bioavailability of NMN, representing a promising nanocarrier strategy for future therapeutics targeting neurodegenerative diseases.

## 4 Conclusion

This study involved the development of a novel, brain-targeted NMN delivery system utilizing surface-modified, biodegradable PEG-PLGA lipid nanoparticles functionalized with Lf-targeted ligands. The prepared LNPs exhibited a spherical morphology, a small particle size, a uniform distribution, and a negatively charged surface. *In vitro* experiments demonstrated that both NMN-LNPs and NMN-LNPs-Lf exhibited low cytotoxicity. Cell uptake assays revealed that NMN-LNPs and NMN-LNPs-Lf significantly increased intracellular NMN levels compared to free NMN. *In vivo* distribution studies further revealed that Lf-modified encapsulated LNPs significantly enhanced NMN’s targeting of the brain. These findings suggest that this delivery system has the potential to achieve brain-targeted NMN delivery and provide a foundation for subsequent research. However, it should be noted that the therapeutic efficacy in neurodegenerative disease models requires further validation. Future studies should incorporate animal models to evaluate functional improvements and relevant pathological indicators, thereby providing a more comprehensive understanding of its potential applications.

## Data Availability

The original contributions presented in the study are included in the article/supplementary material, further inquiries can be directed to the corresponding authors.
